# Allergic reaction and metal hypersensitivity after shoulder joint replacement

**DOI:** 10.1007/s12306-021-00729-4

**Published:** 2021-11-01

**Authors:** A. Pautasso, I. Zorzolo, E. Bellato, P. Pellegrino, A. Ferrario, E. Pira, F. Castoldi

**Affiliations:** 1 University of Turin, Via Gianfranco Zuretti 29, 10126 Turin, Italy; 2 Orthopaedic and Traumatology Department, San Luigi Gonzaga Hospital, University of Turin, Regione Gonzole 10, 10043 Orbassano, Italy; 3 Oncologic Orthopaedic Department, Orthopaedic and Trauma Center – Città della Salute e della Scienza, University of Turin, Via Gianfranco Zuretti 29, 10126 Turin, Italy; 4 Occupational Medicine Division, Department of Public Health and Pediatric Sciences, Orthopaedic and Trauma Center – Città della Salute e della Scienza, University of Turin, Via Gianfranco Zuretti 29, 10126 Turin, Italy

**Keywords:** Patch test, Allergies, Metal hypersensitivity, Shoulder arthroplasty, Cement

## Abstract

**Purpose:**

Metal ion release may cause local and systemic effects and induce hypersensitivity reactions. The aim of our study is first to determine if implant-related hypersensitivity correlates to patient symptoms or not; second, to assess the rate of hypersensitivity and allergies in shoulder arthroplasty.

**Methods:**

Forty patients with shoulder replacements performed between 2015 and 2017 were studied with minimum 2-year follow-up; no patient had prior metal implants. Each patient underwent radiographic and clinical evaluation using the Constant-Murley Score (CMS), 22 metal and cement haptens patch testing, serum and urine tests to evaluate 12 metals concentration, and a personal occupational medicine interview.

**Results:**

At follow-up (average 45 ± 10.7 months), the mean CMS was 76 ± 15.9; no clinical complications or radiographic signs of loosening were detected; two nickel sulfate (5%), 1 benzoyl peroxide (2.5%) and 1 potassium dichromate (2.5%) positive findings were found, but all these patients were asymptomatic. There was an increase in serum aluminum, urinary aluminum and urinary chromium levels of 1.74, 3.40 and 1.83 times the baseline, respectively. No significant difference in metal ion concentrations were found when patients were stratified according to gender, date of surgery, type of surgery, and type of implant.

**Conclusions:**

Shoulder arthroplasty is a source of metal ion release and might act as a sensitizing exposure. However, patch test positivity does not seem to correlate to hypersensitivity cutaneous manifestations or poor clinical results. Laboratory data showed small constant ion release over time, regardless of gender, type of shoulder replacement and implant used.

**Levels of evidence:**

Level II.

## Introduction

Contact dermatitis is a common disease affecting up to 20% of the general population. Metals represent the most common allergens. In particular, nickel (Ni) sensitivity has been reported to have a prevalence of 11.4%, followed by cobalt (Co) at 2.7% and chromium (Cr) at 1.8% [1]. Other metals known to cause allergies are beryllium (Be), tantalum (Ta), titanium (Ti) and vanadium (V) [2–4]. All these metals are used for the production of orthopedic implants [5, 6]. Moreover, components of the bone cement such as polymethyl methacrylate (PMMA), benzoyl peroxide (BPO), N,N-dimethyl-p-toluidine, and hydroquinone [7] have been implicated as relevant allergens.

Allergy complications subsequent to the use of orthopedic implants encompass both skin reactions (e.g., local [4, 8, 9] and systemic eczema [4, 7–10], urticaria [9, 11], sterile fistulas [12]) and non-cutaneous manifestations, both systemic (e.g., chronic fatigue syndrome, fibromyalgia and autoimmune/inflammatory syndrome [13]) and local (pain, joint effusion and reduced range of motion [7, 9, 14]).

The role of immunologic reactions in implant failure has not been fully understood. In addition, the effect of pre-existing metal allergies resulting in implant failure and the role of metal released by the implant on secondary sensitization are unknown [5].

All implants undergo corrosion inside the human body by various mechanisms [6], and elevated concentrations of metal ions have been measured in capsular and periprosthetic tissues [15, 16], blood [15, 17], urine [17], and in distant organs including liver, spleen, and lymph nodes [18].

Most of the literature on metal hypersensitivity is focused on total hip (THA) [15, 19, 20] and total knee arthroplasty (TKA) [4, 21], due to their widespread implantation and, partially, due to the high complication rate of the first generation of metal-on-metal hip coupling [2, 16, 17].

Shoulder arthroplasty is also prone to corrosion, as demonstrated by the increase in metal ions in blood [22] and urine [23] after surgery. Implant retrieval studies show that tribocorrosion, a combination of fretting and corrosion marks, can occur at taper junctions of modular components of shoulder prosthesis[24–26]. Moreover, many taper junctions connect components made of different metal alloys, resulting in the implant being subjected to galvanic corrosion [27]. Additionally, metal debris can be released from unexpected metal-on-metal contacts, such as in chronic implant instability [28] or in the case of retained metal glenoid anchor emerged at the joint line [29].

Despite its constant incidence increase in the past decade [30], there is a paucity of evidence on the effect of metal and bone cement hypersensitivity in shoulder arthroplasty. To our knowledge, few studies have been published on this subject [9, 31–33].

The aim of our study is: first, to determine if implant related hypersensitivity correlates to patient symptoms or not; second, to assess the prevalence of implant hypersensitivity and the allergens that may trigger the reaction in shoulder arthroplasty.

## Materials and methods

A retrospective monocentric study was conducted with prospective data collection. In light of the Italian law, no institutional review board approval was mandatory for this study. The study has been performed in accordance with the ethical standards of the 1964 Declaration of Helsinki and has been carried out in accordance with relevant regulations of the Italian National Health Care System. Informed consent was obtained from all patients.

One hundred and sixteen primary shoulder arthroplasties were performed at CTO Hospital - Città della Salute e della Scienza - Turin, Italy between January 2015 and December 2017. The inclusion criteria were glenohumeral osteoarthritis (either concentric or cuff tear arthropathy) and proximal humeral fractures treated with shoulder arthroplasty with a minimum follow-up of 2 years. Exclusion criteria were: the presence or the history of other implants besides the shoulder replacement (e.g., other orthopedic devices, cardiac and dental implants, etc.), immunological disorders or immunotherapy, megaprosthesis, and pregnancy.

All the patients included in the study underwent a radiographic and clinical evaluation performed by the same orthopedic surgeon [AP] at follow-up. Clinical shoulder function was assessed with the Constant-Murley Score (CMS) [34], and x-rays were reviewed to rule out any sign of loosening and incongruity of the prosthetic components.

An occupational medicine evaluation was conducted along with a questionnaire about hypersensitivity and environmental exposure to sensitizing compounds. Serum, urine tests and a patch test for metal ions were performed. Serum and urine samples were analyzed by inductively coupled plasma mass spectrometry (ICP-MS). The obtained data were compared with the maximum reference values according to the Italian Society Reference Values (SIVR) [35] and Italian Higher Institute of Health’s report [36]. The haptens used in the patch test and their concentration, and the metals tested in the serum and urine samples are reported in Table 1. The patients’ skin was checked for reactions by the same senior occupational physician [AF] at 48 and 72 h after the patch test application. Results were recorded according to the Italian Society of Environment and Occupational Allergological Dermatology (SIDAPA) criteria [37].

**Table 1:** Metal and cement components tested with the patch test

#	Metals tested	Concen-tration	ID	#	Substances tested	Concen-tration	ID
1	Aluminum Hydroxide *	10%	99,994	17	(2-Hydroxyethyl) Methacrylate	1%	E2477
2	Ammonium Molybdate	1%	2401X	18	Benzoyl Peroxide	1%	E0201
3	Cadmium Sulfate *	2%	2307X	19	Ethyleneglycol Dimethacrylate	2%	E1850
4	Cobalt Chloride *	1%	E002	20	Hydroquinone	1%	E0800
5	Cobalt (II) Sulfate	1%	0615X	21	Methyl Methacrylate	2%	E1800
6	Chromium (III) Chloride *	2.5%	2412X	22	Triethyleneglycol Methacrylate	2%	E1851
7	Chromium Sulfate	0.2%	1833X				
8	Iron Chloride *	2%	2406				
9	Iron Sulfate	2%	2405				
10	Manganese Bi-oxide *	10%	99,997				
11	Nickel Sulfate *	5%	E0003				
12	Potassium Dichromate	0.5%	E0001				
13	Tantalum *	1%	2311X				
14	Titanium (IV) Oxide *	0.1%	2419X				
15	Vanadium Chloride *	0.2%	2449X				
16	Zirconium (IV) Sulfate *	2.5%	1019X				

*: Serum and urine metal concentrations analyzed with ICP-MS; ID: International product identification

Statistical analysis was performed using IBM SPSS® (Data Analysis and Statistical Software). The Kolmogorov–Smirnov test of normality was used to study the values distributions of all data series obtained from CMS and ion concentrations analysis. All series had a non-normal distribution; as a consequence, nonparametric tests such as the Mann–Whitney U test and the Kruskal–Wallis test were used to compare the different subgroups of patients’ values (stratification of the samples by gender, surgical date, type of arthroplasty performed, and implant used). P values of < 0.05 were considered to be significant.

## Results

Forty-nine patients were eligible to be included, but only 40 patients agreed to be enrolled in our study. The mean age was 69 ± 7 years old (53–78), 27 (67.5%) patients were female and 13 (32.5%) were male. Patients’ characteristics stratified by diagnosis, type of arthroplasty performed, surgical date, occupational status, history of hypersensitivity, implant used and its metal composition are reported in Table 2 and Table 3. The mean follow-up was 45 ± 10.7 months (27–60).

**Table 2:** Demographic sample description

Characteristics	No. Patients	%
*Preoperative diagnosis*		
Proximal humeral fracture	26	65.0%
Gleno-humeral osteoarthritis	14	35.0%
*Type of replacement*		
Partial (Hemiarthroplasty)	15	37.5%
Total (Revers Shoulder Arthroplasty)	25	62.5%
*Type of shoulder prosthesis*		
Bigliani/Flatow® – Zimmer Biomet©	13	32.5%
Tornier Aequalis™ FX – Wright Medical Group©	11	27.5%
Tornier Aequalis™ Reversed II – Wright Medical Group©	16	40.0%
Cemented implants (out of all arthroplasty)	19	47.5%
*Year of surgery*		
2015	17	42.5%
2016	12	30.0%
2017	11	27.5%
*Employment*		
Active	9	22.5%
Retired	31	77.5%
*Current/Previous sector business*		
Industry	8	20.0%
Service	32	80.0%
Patients with allergic or suspected symptoms	16	40.0%
Skin Manifestation (out of all patients investigated)	13	32.5%

**Table 3:** Prosthetic metallic components

Implant		Humeral stem	Humeral head	Humeral bearing	Glenosphere	Baseplate	Screws
Bigliani/Flatow®– Zimmer Biomet©	Cemented or not	Ti-6Al-4 V + Ta coated	/	UHMWPE	Cr-Co-Mo alloy	Ti-6Al-4 V + Ta coated	Ti-6Al-4 V
Tornier Aequalis™ Reversed II – Wright Medical Group©	Cemented	Cr-Co alloy	/	UHMWPE	Cr-Co alloy	Ti-6Al-4 V + HA coated	Ti-6Al-4 V
Tornier Aequalis™ Reversed II – Wright Medical Group©	Not Cemented	Ti-6Al-4 V	/	UHMWPE	Cr-Co alloy	Ti-6Al-4 V + HA coated	Ti-6Al-4 V
Tornier Aequalis™ FX – Wright Medical Group©	Cemented or not	Ti-6Al-4 V + HA coated	Cr-Co alloy	/	/	/	/

Ti: Titanium; Al: Aluminum; V: Vanadium; Ta: Tantalum; Cr: Chromium; Co: Cobalt; Mo: Molybdenum; UHMWPE: Ultra-High-Molecular-Weight Polyethylene; HA: Hydroxyapatite

The mean CMS recorded at follow-up was 76 ± 15.9 (43–97). In the proximal humeral fracture group the mean CMS was 75 ± 16.2 (43–97), while in the osteoarthritis group the CMS was 77 ± 15.8 (47–97) (p = 0.69). None of the patients reported either local or systemic skin reactions, pruritus, joint effusion or prolonged joint pain at rest. No radiographic signs of loosening or other complications were detected at final follow-up.

The occupational medicine questionnaire highlighted eventual sensitizing environmental exposure in 17 patients. Eight patients were industrial factory employees with potential for contact with allergenic substances (e.g. paints, solvents, vegetables, mineral and industrial oils, resins); in particular 3 of these were metalworkers. Out of the 32 patients who worked in the service sector, 4 reported contacts with metal substances and 5 further patients reported possible contacts with other allergenic materials in their professional activity.

In 16 (40%) patients allergic symptoms were detected, either formally diagnosed or self-reported. Four of these patients reported intolerance, without prior investigation, to costume jewelry, with the appearance of erythema in the contact area even before shoulder replacement was performed.

Patch tests were positive in 4 patients (10%). Three cases were mildly positive: 1 patient (2.5%) to nickel, 1 (2.5%) to BPO and 1 (2.5%) to potassium dichromate. Another 1 case (2.5%) was found strongly positive to nickel (patch-test results are summarized in Table 4). The 2 cases of nickel hypersensitivity were among the ones who reported dermal reactions to costume jewelry. The patient mildly positive to BPO underwent a cemented shoulder replacement. Despite the patch test positivity, these 4 patients did not show any clinical or radiographic signs of shoulder implant complications (Patients 9, 13, 24 and 28 in Appendix 1).

**Table 4:** Prosthetic metallic components

Haptens tested	Positive reactions (%)	Patch test reading (%)			
		** + + + **	** + + **	** + **	** ± **
(2-Hydroxyethyl) Methacrylate	0	0	0	0	0
Aluminum Hydroxide	0	0	0	0	0
Ammonium Molybdate	0	0	0	0	0
Benzoyl Peroxide	1 (2.5%)	0	0	1 (2.5%)	0
Cadmium Sulfate	0	0	0	0	0
Cobalt Chloride	0	0	0	0	0
Cobalt (II) Sulfate	0	0	0	0	0
Chromium (III) Chloride	0	0	0	0	0
Chromium Sulfate	0	0	0	0	0
Ethyleneglycol Dimethacrylate	0	0	0	0	0
Iron Chloride	0	0	0	0	0
Iron Sulfate	0	0	0	0	0
Hydroquinone	0	0	0	0	0
Manganese Bi-oxide	0	0	0	0	0
Methyl Methacrylate	0	0	0	0	0
Nickel Sulfate	2 (5%)	1 (2.5%)	0	1 (2.5%)	0
Potassium Dichromate	1 (2.5%)	0	0	1 (2.5%)	0
Tantalum	0	0	0	0	0
Titanium (IV) Oxide	0	0	0	0	0
Triethyleneglycol Methacrylate	0	0	0	0	0
Vanadium Chloride	0	0	0	0	0
Zirconium (IV) Sulfate	0	0	0	0	0

± : Slight erythema; + : Uniform erythema with edema, papules or slight vesicles possible; +  + : Erythema, edema, evident papules and vesicles that can protrude from the patch application area; +  +  + : Erythema, edema, very evident papules and vesicles, sometimes confluent in bubbles [29]

**Fig. 1:**
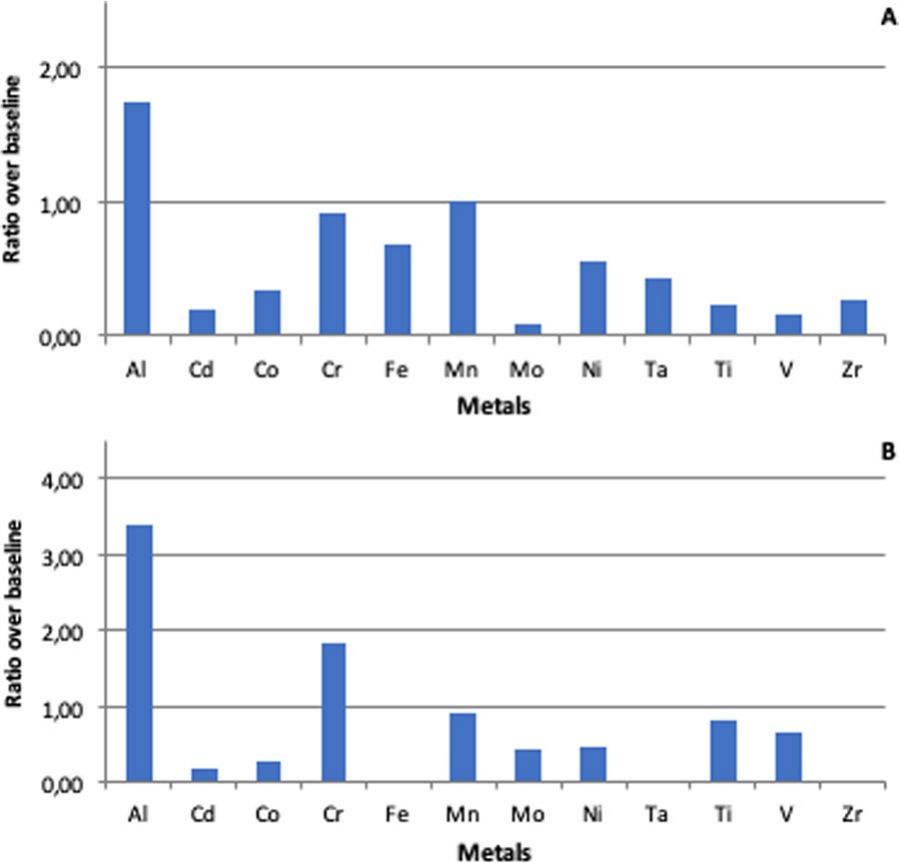
Ratio between Observed Average Values (OAV) of each metal and its Reference Maximum Value (RMV) detected in serum (fig. **A**) and urine (fig. **B**). On the y-axis, the ratio expressed as a number over each metal baseline: a value over 1.00 is not physiological because over its maximum reference

Serum metal ion ICP-MS analysis showed an increase in aluminum concentration with a mean value of 10.43 ± 3.45 μg/L (4.80–21.30; Reference Maximum Values [RMV]: 6.00), which is 1.74 times the baseline. The other serum metal ion concentrations were in range of normality (Fig. 1a, Table 5). Regarding the urinary metal ion ICP-MS analysis, a 3.40-fold and a 1.83-fold increase over aluminum and chromium RMV were observed, respectively (urinary aluminum mean value 20.38 ± 12.52 μg/L [5.84–61.20; RMV: 6.00]; urinary chromium mean value 0.64 ± 0.30 μg/L [0.18–1.70; RMV: 0.35]) (Fig. 1b, Table 5).

**Table 5:** Serum and urine laboratory results. Data expressed in µg/l, except iron (Fe) values expressed in mg/l

Serum laboratory results						Urine laboratory results				
Average		sSD	Min	Max	RMV	Average	sSD	Min	Max	RMV
Al	10.43	3.45	4.80	21.30	6.00	20.38	12.52	5.84	61.20	6.00
Cd	0.30	0.17	0.10	1.20	1.50	0.26	0.43	0.00	2.60	1.50
Co	0.34	0.27	0.18	1.85	1.00	0.43	0.48	0.01	1.80	1.50
Cr	0.46	0.14	0.26	0.96	0.50	0.64	0.30	0.18	1.70	0.35
Fe	444.85	58.77	303.00	508.00	646.49	Not detected				Not available
Mn	7.99	1.77	3.20	12.80	8.00	3.63	7.41	0.22	41.88	4.00
Mo	0.41	0.74	0.10	4.60	5.00	42.32	33.36	4.01	172.21	100.00
Ni	1.12	0.68	0.53	4.02	2.00	2.34	2.21	0.24	9.65	5.00
Ta	0.14	0.10	0.00	0.32	0.32	Not detected				0.28
Ti	16.85	3.25	10.97	25.77	70.00	8.25	4.90	1.32	20.94	10.00
V	0.03	0.03	0.00	0.18	0.20	0.65	0.30	0.16	1.36	1.00
Zr	0.26	0.17	0.16	1.22	0.95	Not detected				2.00

sSD: sample Standard Deviation; Min: Minimum value; Max: Maximum value; RMV: Reference Maximum Value; Al: Aluminum; Cd: Cadmium; Co: Cobalt; Cr: Chromium; Fe: Iron; Mn: Manganese; Mo: Molybdenum; Ni: Nickel; Ta: Tantalum; Ti: Titanium; V: Vanadium; Zr: Zirconium

No significant difference in serum nor urine metal ion concentrations was found when patients were stratified according to gender (p ≥ 0.17), year of surgery (p ≥ 0.14), type of surgery (Hemiarthroplasty vs Reverse Shoulder Replacement) (p ≥ 0.31), and type of implant (Bigliani/Flatow®–Zimmer Biomet© vs Tornier Aequalis™ FX–Wright Medical Group© vs Tornier Aequalis™ Reversed II–Wright Medical Group©) (p ≥ 0.14).

Patients’ details of serum and urinary levels of metal ions are to be found in Appendix 2.

## Discussion

The purpose of this study was, first, to evaluate whether allergies, diagnosed with a patch test, were related or not to patients’ symptoms after shoulder arthroplasty; secondly, to determine the prevalence of implant-related hypersensitivity and the allergens that could trigger this reaction.

Hypersensitivity following shoulder arthroplasty is an underexplored subject in the literature. Only a few studies have focused on the possible allergic reactions to metals in patients who underwent shoulder replacement [32, 33]. Ko et al. [32] identified 6 patients with metal allergy after shoulder arthroplasty in a population of 1243 patients. Most of them had a routine early post-operative course; however, a progressive worsening in pain and range of motion, without any cutaneous manifestation, appeared within the first 12 weeks. After infection was ruled out, patients were investigated for hypersensitivity: 3 resulted allergic to nickel, 1 to cobalt, 1 to chromium and 1 to multiple metals (Co, Cr, Mo, Ti, and Al). All the patients but the latter one (no hypoallergenic implant was available for him) underwent revision surgery and 4 reported improvement in symptoms. Kennon et al. [33] have recently retrospectively reported on 43 patients, with a total of 52 having undergone shoulder arthroplasty, who self-reported metal hypersensitivity. Patients referred allergies to nickel (37 cases), cobalt chrome (4 cases), copper (2 cases), gold (2 cases), titanium (1 case), zinc (1 case), and nonspecific metals (8 cases). Eight patients reported multiple metal allergies. A subset of 13 shoulders underwent skin patch testing, and only one resulted negative. Despite the high prevalence of nickel sensitivity reported in patients’ past medical history before surgery, all patients received an implant containing this metal. Nevertheless, they reported a significant improvement in function and pain relief after surgery, without any difference between the group with a positive skin patch and the patients that only declared a metal hypersensitivity. There were 2 insidious glenoid loosenings, not attributable to hypersensitivity and no cutaneous reactions.

Additionally, non-metallic materials can play a role in allergic reactions in patients with shoulder replacement. Bircher et al. [9] reported on a patient with cemented total shoulder arthroplasty who developed chronic pain associated with an erythematous plaque on the upper arm characterized by lymphocytic infiltrate. After BPO hypersensitivity was diagnosed with skin patch testing, the patient underwent an uncemented revision procedure that led to the resolution of the symptoms.

In our study the rate of metal and bone cement hypersensitivity was 10% (4 patients). Nickel was the commonest allergen, as described in the general population[1]. The two patients that were sensitive to nickel received a reverse arthroplasty containing this metal in the glenosphere. Interestingly, both reported preoperative skin reactions to costume jewelry, and thus, the shoulder implant was not the sensitizing exposure. One patient with shoulder hemiarthroplasty characterized by a humeral head made of chromium proved to be hypersensitive to potassium dichromate and was found to have increased serum and urine levels of chromium. He was previously exposed to metals in his work; however, he never experienced skin hypersensitivity symptoms. The last skin patch was positive to BPO, a normally used initiator in bone cement [7]. This patient’s medical history was negative for previous exposures and a cemented shoulder arthroplasty was performed, which could be the sensitizing factor. However, BPO is a well-known skin irritant, so a weak positivity, as occurred in this case, can be subsequent either to contact sensitization or just to irritation [38].

Although 4 patients had a positive patch test, they experienced neither skin reactions nor non-cutaneous manifestations connected to metal and bone cement hypersensitivity. At the latest follow-up, x-rays showed no signs of failure and good results in terms of CMS (Patients 62, 81, 84 and 96 in Appendix 1) were observed.

Our metal ion ICP-MS analysis showed an increase in serum and urinary aluminum and urinary chromium. However, no significant correlation could be found, especially with hypersensitive and exposed subjects since we could identify only one.

Unlike Reiner et al.’s work [22], we could not find a difference in ion release between hemiarthroplasty and reverse shoulder arthroplasty. In their study, titanium blood concentration was significantly higher in the reverse shoulder arthroplasty group, possibly due to galvanic corrosion at the cobalt-chromium glenosphere and at the titanium alloy baseplate taper junction. Also, 4 titanium screws were used to secure the baseplate, which might have acted as an additional source for titanium ion release. The semiconstrained biomechanical concept of the reverse shoulder replacement design places high friction and shear forces at the glenosphere-baseplate and at the baseplate-screws-bone interfaces. Increased micro-motions might facilitate fretting and corrosion damage resulting in higher blood metal ion concentrations in these patients. Furthermore Reiner et al. [22] found significantly higher cobalt, chromium and titanium levels in total shoulder arthroplasty patients than in controls without any implant. A direct comparison with our study cannot be drawn since their measurements were taken on whole blood, resulting in lower values than ours [39].

Urinary metal ion concentrations have been reported by Khan et al. [23] to be increased in shoulder arthroplasty. They found an increase in excretion of cobalt, chromium, aluminum, titanium and molybdenum unlike the sole rise in chromium and aluminum we detected. However, their results were based on a single case report of a patient with shoulder metallosis.

Aluminum is a component of the titanium alloy; however, we found an isolated increase in aluminum with mean titanium concentration below the RMV. Studies that tested aluminum concentrations found no significant increase both in hip (both in metal-on-metal [40–42] and ceramic-on-ceramic coupling [41, 42]) and in knee replacement [43]. Chronic aluminum exposure has been implied in a specific encephalopathy with a dementia syndrome. Serum concentration of approximately 13 μg/L measured in occupationally-exposed workers correlates with a decline in neuropsychological tests; however, those subjects did not manifest encephalopathy at long-term follow-up [44]. The mean data we reported are below this threshold. The measurement of aluminum concentration is complicated by its environmental abundance and the small amount in serum, placing this analysis at high risk of contamination [45].

We did not find any difference in ion release over time when we stratified our patients according to the length of follow-up. In hip arthroplasty with metal-on-metal coupling, metal ion release is greater at a short follow-up resulting in higher cobalt and chromium whole blood or serum and urine concentrations [17, 40, 46]. This phenomenon has been attributed to an accelerated wear rate that affects metal implants when they are subjected to the early load cycles; this elevated wear rate decreases over time with the running-in of the components [47]. The reduction in wear rate translates clinically to a significant reduction in cobalt and chromium concentrations in urine and whole blood or serum at a longer follow-up, despite remaining higher than control groups [17, 40]. Shoulder arthroplasty is subjected to accelerated running-in wear as well [48]; however, the typical metal-on-polyethylene coupling used in shoulder replacement might lead to a lower release of metal ions. It may be possible that we were not able to identify an ion concentration trend over time due to the lack of multiple measurements in the same patient.

### Limitations and strengths

This study had several limitations. First, we could include only a small number of patients and this also affected patients’ stratification process. Second, we did not have a control group without implants. Third, we did not have preoperative data on skin patch testing and on metal ion concentrations, despite data being collected prospectively. Fourth, we could not draw definitive conclusions about the causative link between shoulder arthroplasty and hypersensitivity since we had to rely on patients’ past medical history for previous metal sensitizations and exposure. In the literature [49] there is no general consensus on the role of patch tests before surgery, due to its intrinsic allergenic potentiality and lack of long-term impact on implants survival (e.g., Bravo et al. [21] reported no difference in joint revision rates among patients who underwent knee arthroplasty with a positive or a negative preoperative patch test). Fifth, we were able to collect serum and urine samples during a single follow-up evaluation only; therefore, we cannot describe a precise trend of ion release over time. Sixth, we used skin patch tests, which represent the gold standard for contact dermatitis diagnosis, since they are easy to use, readily available and cheap. However, other in-vitro tests are available such as the Lymphocyte Transformation Test (LTT) that measures lymphocytes in peripheral blood after allergen exposure [49]. LTT is more appropriate for diagnosing metal hypersensitivity induced by deep implants and it is a better option for detecting systemic allergies, while patch test better identifies cutaneous hypersensitivity [50]. However, skin patch tests are cheaper, widely available, and have good reliability.

Despite these critical points, to our knowledge this is the first study that provides data on allergies and hypersensitivity of patients undergoing shoulder replacement. This study explores hypersensitivity, allergies to metals or bone cement components in patients with shoulder replacement using patch tests. Unlike prior studies in the literature that only investigated patients formally diagnosed or self-reported metal allergies undergoing shoulder replacement, this cohort of patients treated with shoulder arthroplasty were studied regardless of their immunological status before and after surgery.

Additionally, the time point for which patients underwent blood and urine metal ion tests was different from patient to patient (i.e., some were tested at 1 year, some at 2 years after surgery). Post-hoc analysis of this data allowed us to understand the rate of ion release in relation to the length of follow-up.

## Conclusions

Shoulder arthroplasty is a possible source of metal ion release and, along with other substances such as BPO, are potential causes of hypersensitivity. In this study, the rate of patch test positivity was 10%, but this does not correlate to cutaneous manifestations or poor clinical and radiographic results. Systemic ion release over time is a possible concern, in particular regarding aluminum and chromium, but no clinical effects were observed in our study group. Factors like gender, type of shoulder replacement and implant used did not play a role either in hypersensitivity or in systemic ion release for this study.

## Data Availability

Not applicable.

## Appendix 1

**Table:**

Id	Dos	Type of surgery	Type of Implant	Cemented Implant?	CMS	Current/Previous sector business	Known Or suspected allergic symptoms	Patch test result
1	Jan-15	RSA	Bigliani/Flatow® – Zimmer Biomet©	No	47	Service	No	Negative
2	Feb-15	RSA	Tornier Aequalis™ Reversed II – Wright Medical Group©	No	97	Service	No	Negative
3	Mar-15	RSA	Tornier Aequalis™ Reversed II – Wright Medical Group©	No	70	Service	No	Negative
4	Apr-15	RSA	Tornier Aequalis™ Reversed II – Wright Medical Group©	No	43	Service	Yes, with skin manifestation	Negative
5	Dec-15	RSA	Bigliani/Flatow® – Zimmer Biomet©	No	83	Industry	No	Negative
6	Sep-15	RSA	Tornier Aequalis™ Reversed II – Wright Medical Group©	No	86	Service	Yes, without skin manifestation	Negative
7	Jan-16	Hemiarthroplasty	Tornier Aequalis™ FX – Wright Medical Group©	Yes	52	Service	Yes, with skin manifestation	Negative
8	Mar-16	Hemiarthroplasty	Tornier Aequalis™ FX – Wright Medical Group©	Yes	77	Service	Yes, with skin manifestation	Negative
9	May-16	Hemiarthroplasty	Tornier Aequalis™ FX – Wright Medical Group©	Yes	81	Industry	No	Benzoyl Peroxide ( +)
10	Apr-15	Hemiarthroplasty	Tornier Aequalis™ FX – Wright Medical Group©	No	90	Industry	No	Negative
11	Sep-16	RSA	Tornier Aequalis™ Reversed II – Wright Medical Group©	Yes	65	Service	No	Negative
12	Apr-16	RSA	Bigliani/Flatow® – Zimmer Biomet©	Yes	69	Service	Yes, with skin manifestation	Negative
13	Dec-15	Hemiarthroplasty	Tornier Aequalis™ FX – Wright Medical Group©	Yes	84	Service	Yes, without skin manifestation	Potassium Dichromate ( +)
14	Oct-16	Hemiarthroplasty	Bigliani/Flatow® – Zimmer Biomet©	Yes	67	Service	No	Negative
15	Nov-16	Hemiarthroplasty	Tornier Aequalis™ FX – Wright Medical Group©	No	79	Service	Yes, with skin manifestation	Negative
16	Feb-17	RSA	Bigliani/Flatow® – Zimmer Biomet©	No	51	Industry	No	Negative
17	May-16	RSA	Bigliani/Flatow® – Zimmer Biomet©	No	49	Service	Yes, with skin manifestation	Negative
18	Dec-15	Hemiarthroplasty	Bigliani/Flatow® – Zimmer Biomet©	No	77	Service	No	Negative
19	Jul-17	RSA	Bigliani/Flatow® – Zimmer Biomet©	No	79	Service	Yes, with skin manifestation	Negative
20	Apr-17	RSA	Tornier Aequalis™ Reversed II – Wright Medical Group©	No	89	Service	Yes, with skin manifestation	Negative
21	May-17	RSA	Tornier Aequalis™ Reversed II – Wright Medical Group©	Yes	85	Service	No	Negative
22	Feb-16	RSA	Tornier Aequalis™ Reversed II – Wright Medical Group©	No	89	Industry	Yes, with skin manifestation	Negative
23	Apr-15	RSA	Tornier Aequalis™ Reversed II – Wright Medical Group©	No	68	Service	No	Negative
24	Aug-17	RSA	Tornier Aequalis™ Reversed II – Wright Medical Group©	Yes	62	Service	Yes, with skin manifestation	Nickel Sulfate (+ + +)
25	Jul-17	Hemiarthroplasty	Bigliani/Flatow® – Zimmer Biomet©	Yes	45	Service	No	Negative
26	Jan-16	RSA	Tornier Aequalis™ Reversed II – Wright Medical Group©	Yes	95	Service	No	Negative
27	Oct-17	RSA	Tornier Aequalis™ Reversed II – Wright Medical Group©	Yes	84	Industry	No	Negative
28	Oct-17	RSA	Bigliani/Flatow® – Zimmer Biomet©	Yes	96	Industry	Yes, with skin manifestation	Nickel Sulfate ( +)
29	May-16	RSA	Tornier Aequalis™ Reversed II – Wright Medical Group©	No	63	Service	No	Negative
30	Apr-16	Hemiarthroplasty	Tornier Aequalis™ FX – Wright Medical Group©	No	92	Service	No	Negative
31	Mar-17	RSA	Tornier Aequalis™ Reversed II – Wright Medical Group©	Yes	91	Service	No	Negative
32	Feb-15	RSA	Tornier Aequalis™ Reversed II – Wright Medical Group©	No	86	Service	Yes, without skin manifestation	Negative
33	May-15	Hemiarthroplasty	Tornier Aequalis™ FX – Wright Medical Group©	Yes	85	Service	Yes, with skin manifestation	Negative
34	Dec-15	Hemiarthroplasty	Tornier Aequalis™ FX – Wright Medical Group©	Yes	74	Service	No	Negative
35	Nov-15	Hemiarthroplasty	Tornier Aequalis™ FX – Wright Medical Group©	Yes	49	Service	No	Negative
36	Sep-15	RSA	Bigliani/Flatow® – Zimmer Biomet©	No	77	Service	No	Negative
37	Jun-15	Hemiarthroplasty	Tornier Aequalis™ FX – Wright Medical Group©	Yes	71	Service	No	Negative
38	Oct-17	Hemiarthroplasty	Bigliani/Flatow® – Zimmer Biomet©	No	97	Industry	Yes, with skin manifestation	Negative
39	Aug-17	RSA	Tornier Aequalis™ Reversed II – Wright Medical Group©	No	90	Service	No	Negative
40	Jan-15	RSA	bigliani/Flatow® – Zimmer Biomet©	Yes	88	Service	No	Negative

Detailed information of each patient included in the study (DOS: date of surgery; RSA: Reverse Shoulder Arthroplasy; CMS: Costant Murley Score)

## Appendix 2

**Table:**

ID	Serum test results												Urine test results											
	Al	Cd	Co	Cr	Fe	Mn	Mo	Ni	Ta	Ti	V	Zr	Al	Cd	Co	Cr	Fe	Mn	Mo	Ni	Ta	Ti	V	Zr
1	16.80	0.20	0.22	0.33	315.00	5.60	0.16	0.61	0.17	11.92	0.02	0.19	14.20	0.01	0.92	0.67	0.00	2.35	16.04	0.91	0.00	2.36	0.48	0.00
2	8.60	0.41	0.27	0.57	457.00	8.70	0.30	1.28	0.20	13.85	0.03	0.23	19.90	2.60	0.29	0.82	0.00	1.43	76.90	9.65	0.00	16.80	0.33	0.00
3	6.80	0.61	0.32	0.48	507.00	9.60	0.22	0.88	0.18	16.04	0.04	0.21	54.30	0.74	1.03	0.80	0.00	3.17	65.43	3.46	0.00	14.17	0.75	0.00
4	10.40	0.40	0.37	0.49	452.00	6.40	0.34	1.12	0.21	14.08	0.02	0.21	15.20	0.30	1.73	0.79	0.00	2.21	4.48	5.00	0.00	4.99	0.99	0.00
5	11.80	0.29	0.29	0.44	502.00	9.20	0.26	0.97	0.17	15.48	0.03	0.22	29.30	0.12	0.08	0.88	0.00	0.40	25.55	1.37	0.00	4.87	0.65	0.00
6	8.40	0.19	0.18	0.37	303.00	8.80	0.14	0.63	0.12	10.97	0.01	0.16	16.00	0.08	0.01	0.45	0.00	0.55	46.46	0.35	0.00	4.49	0.32	0.00
7	10.60	0.21	0.23	0.30	411.00	7.00	0.18	0.91	0.16	15.17	0.02	1.22	29.70	0.08	1.52	0.30	0.00	25.86	15.84	4.63	0.00	7.72	0.55	0.00
8	9.00	0.24	0.48	0.42	390.00	7.90	0.24	4.02	0.20	13.97	0.02	0.20	21.90	0.02	0.37	1.21	0.00	5.28	114.98	1.74	0.00	20.94	0.76	0.00
9	9.10	0.24	0.22	0.42	498.00	6.80	0.30	0.88	0.15	16.00	0.02	0.21	16.90	0.00	0.10	0.68	0.00	1.12	35.33	5.63	0.00	7.93	0.69	0.00
10	16.20	0.34	0.50	0.47	496.00	8.50	0.20	0.94	0.00	18.52	0.03	0.24	10.20	0.14	0.02	0.37	0.00	1.95	4.01	1.00	0.00	6.31	0.22	0.00
11	8.30	0.28	0.72	0.42	484.00	9.20	0.36	0.72	0.22	14.51	0.03	0.22	35.50	0.03	0.08	0.50	0.00	1.07	36.57	0.74	0.00	7.79	0.56	0.00
12	6.30	0.28	0.29	0.37	451.00	7.30	0.20	0.87	0.10	15.09	0.02	0.21	45.20	0.09	0.09	0.48	0.00	0.78	11.54	0.33	0.00	6.68	0.59	0.00
13	18.10	0.31	0.41	0.66	446.00	12.80	0.26	0.82	0.00	18.31	0.03	0.20	22.30	0.03	0.04	0.69	0.00	1.85	58.69	0.92	0.00	7.36	0.97	0.00
14	10.80	0.29	0.26	0.45	432.00	8.30	0.22	0.85	0.00	16.43	0.02	0.23	13.10	0.02	0.02	0.54	0.00	1.18	29.06	1.11	0.00	10.37	0.67	0.00
15	12.20	0.22	0.26	0.35	440.00	7.10	0.16	0.80	0.00	15.24	0.02	0.19	10.90	0.04	0.59	0.46	0.00	5.43	40.20	1.98	0.00	14.59	0.37	0.00
16	21.30	0.45	0.54	0.44	497.00	9.60	0.20	1.03	0.14	16.03	0.03	0.25	20.50	0.57	0.22	0.76	0.00	3.60	20.92	1.87	0.00	6.92	0.93	0.00
17	16.60	0.26	0.36	0.47	395.00	7.80	0.34	3.22	0.00	16.11	0.03	0.21	10.80	0.56	0.82	0.76	0.00	1.69	74.83	1.75	0.00	16.73	0.86	0.00
18	8.40	0.29	0.22	0.89	506.00	10.40	4.60	1.19	0.00	20.15	0.00	0.63	38.40	0.01	0.36	0.52	0.00	0.75	31.08	0.88	0.00	10.84	0.47	0.00
19	9.60	0.20	0.23	0.39	330.00	7.00	0.36	0.87	0.10	15.49	0.02	0.18	17.90	0.04	0.09	0.47	0.00	1.99	19.50	0.92	0.00	2.35	0.37	0.00
20	11.80	0.28	0.31	0.45	445.00	9.10	0.30	2.15	0.14	17.88	0.03	0.21	16.00	0.14	0.06	0.30	0.00	2.54	12.40	0.24	0.00	1.74	0.19	0.00
21	4.80	0.32	0.28	0.46	508.00	9.30	0.27	1.02	0.12	19.94	0.05	0.22	14.80	0.10	1.80	0.56	0.00	41.88	16.38	3.32	0.00	7.65	0.39	0.00
22	10.40	0.31	0.25	0.38	503.00	6.70	0.29	0.88	0.12	19.01	0.02	0.23	9.88	0.17	0.40	0.76	0.00	2.29	50.54	1.06	0.00	6.02	0.93	0.00
23	8.60	0.32	0.30	0.44	486.00	7.60	0.28	0.81	0.22	17.35	0.02	0.26	12.40	0.01	0.19	0.67	0.00	1.95	17.47	9.53	0.00	12.15	0.38	0.00
24	5.40	0.28	0.26	0.33	493.00	7.80	0.58	0.87	0.10	19.49	0.02	0.20	22.30	0.67	0.35	0.93	0.00	6.44	172.21	6.61	0.00	9.77	0.78	0.00
25	11.60	0.31	0.29	0.39	467.00	7.20	0.28	1.17	0.18	18.93	0.02	0.22	8.47	0.09	0.13	0.47	0.00	1.76	50.62	1.29	0.00	7.71	0.79	0.00
26	7.20	0.17	0.18	0.26	332.00	6.20	0.20	0.53	0.00	14.45	0.02	0.19	37.90	0.43	0.21	1.70	0.00	3.62	68.49	2.57	0.00	16.05	1.09	0.00
27	9.60	0.26	0.22	0.33	495.00	7.30	0.44	0.83	0.28	18.59	0.02	0.21	13.40	0.33	0.18	0.55	0.00	2.83	85.82	2.28	0.00	5.00	0.41	0.00
28	10.40	0.28	0.26	0.39	374.00	7.60	0.28	0.95	0.00	19.09	0.02	0.20	61.20	0.21	0.90	1.37	0.00	3.29	91.13	1.14	0.00	16.00	0.81	0.00
29	11.00	0.25	0.33	0.57	426.00	5.30	2.12	1.69	0.00	18.42	0.03	0.18	10.50	0.05	0.10	0.61	0.00	4.60	53.73	1.56	0.00	10.89	1.15	0.00
30	8.60	0.27	0.22	0.38	451.00	6.50	0.24	0.89	0.00	18.12	0.02	0.19	5.84	0.08	0.08	0.18	0.00	0.41	27.31	0.98	0.00	7.63	0.32	0.00
31	8.40	0.30	0.36	0.70	505.00	3.20	0.26	2.18	0.00	25.77	0.18	0.28	15.50	0.25	1.15	0.62	0.00	0.81	44.13	3.14	0.00	15.31	0.59	0.00
32	8.30	0.10	0.40	0.42	409.00	7.10	0.20	0.80	0.20	12.50	0.04	0.20	10.10	0.11	0.59	0.21	0.00	0.70	17.50	1.24	0.00	2.48	1.08	0.00
33	9.80	0.16	0.19	0.48	508.00	8.80	0.40	0.97	0.23	20.20	0.08	0.23	14.10	0.29	0.20	0.42	0.00	1.86	24.30	1.94	0.00	8.45	1.36	0.00
34	10.50	0.12	1.85	0.96	451.00	7.20	0.20	0.81	0.26	21.40	0.07	0.36	21.50	0.60	0.31	0.69	0.00	1.69	25.50	1.80	0.00	5.05	1.12	0.00
35	9.30	0.17	0.20	0.46	433.00	9.00	0.20	0.96	0.22	14.50	0.02	0.16	16.00	0.11	0.10	0.58	0.00	0.55	9.80	0.82	0.00	1.32	0.16	0.00
36	8.80	0.34	0.28	0.55	467.00	10.70	0.20	0.74	0.19	17.00	0.04	0.22	15.30	0.22	0.77	0.52	0.00	2.67	31.70	2.10	0.00	3.70	0.81	0.00
37	10.40	0.48	0.30	0.49	489.00	8.20	0.20	1.08	0.22	16.00	0.03	0.20	8.60	0.13	0.08	0.61	0.00	0.22	48.50	3.03	0.00	6.11	0.96	0.00
38	6.80	0.28	0.18	0.40	365.00	8.10	0.30	0.83	0.24	15.10	0.05	0.25	22.20	0.30	0.23	0.65	0.00	0.80	43.40	1.13	0.00	6.38	0.65	0.00
39	12.90	1.20	0.27	0.43	494.00	12.10	0.10	1.04	0.32	25.60	0.07	0.38	11.10	0.14	0.12	0.25	0.00	0.35	13.00	1.66	0.00	2.87	0.30	0.00
40	13.40	0.18	0.21	0.36	381.00	6.60	0.20	0.84	0.29	11.30	0.03	0.21	26.00	0.31	0.82	0.76	0.00	1.21	61.60	1.85	0.00	3.37	0.34	0.00
